# Exploring the differences in neural oscillation mechanisms before and after sleep deprivation

**DOI:** 10.3389/fnins.2026.1910878

**Published:** 2026-08-03

**Authors:** Zitong Cui, Tiantian Xie, Yueying Zhou, Peiliang Gong, Xiaorui Zhang, Pengpai Wang

**Affiliations:** 1 2011 College, Nanjing Tech University, Nanjing, China; 2 Department of Electrical Engineering, City University of Hong Kong, Hong Kong SAR, China; 3 School of Mathematics Science, Liaocheng University, Liaocheng, China; 4 College of Artificial Intelligence, Nanjing University of Aeronautics and Astronautics, Nanjing, China; 5 College of Computer and Information Engineering, Nanjing Tech University, Nanjing, China

**Keywords:** electroencephalography, individual differences, neural oscillations, psychomotor vigilance task, relative power variability, sleep deprivation, theta oscillations

## Abstract

**Introduction:**

Sleep deprivation is widespread in modern society and can impair cognitive function and behavioral performance. However, the neural changes associated with individual differences in vulnerability to sleep loss remain unclear, particularly whether temporal fluctuations in neural oscillations provide information beyond conventional mean spectral measures.

**Methods:**

We analyzed a publicly available resting-state electroencephalogram (EEG) dataset comprising 64 participants assessed under normal sleep and sleep deprivation conditions, including 28 participants with psychomotor vigilance task (PVT) data. Two oscillatory feature families, mean relative power and the across-epoch variability of relative power, were quantified in the theta, alpha, and beta bands, primarily at the regional level, with channel-level analyses used to further characterize their spatial distribution.

**Results:**

Theta-band features showed the most pronounced changes after sleep deprivation. In particular, theta power variability increased across widespread scalp regions, whereas changes in mean theta relative power were more spatially restricted. Greater increases in theta power variability in the frontal and centro-temporal regions were associated with greater slowing in PVT median reaction time, and the principal association pattern remained evident in sensitivity analyses. Exploratory sex-related analyses identified a significant sex-by-EEG interaction in the centro-temporal region, indicating different linear EEG–PVT slopes between males and females, although the corresponding difference in Spearman correlation strength was not significant.

**Discussion:**

Overall, theta power variability may provide complementary information beyond conventional mean spectral measures and may help characterize individual differences in vigilance decline after sleep deprivation.

## 1 Introduction

Sleep deprivation is common in modern society and has become an important public health concern (Chattu et al., 2018; Ramos et al., 2023). Insufficient sleep can impair daily functioning and increase real-world risks, including reduced work efficiency, attentional failures, and operational errors (Krause et al., 2017; Ramos et al., 2023). These consequences are closely related to impairments across several cognitive domains, including reaction speed, sustained attention, working memory, and executive control (Krause et al., 2017; Lowe et al., 2017; Sen and Tai, 2023). Among these functions, vigilance is particularly sensitive to sleep loss and is commonly assessed using the psychomotor vigilance task (PVT) (Basner et al., 2021; Hudson et al., 2020). Sleep deprivation typically leads to slower responses, more frequent lapses, and less stable behavioral performance (Basner et al., 2021; Hudson et al., 2020). However, the degree of vigilance impairment varies substantially across individuals, with some showing pronounced deterioration and others remaining relatively resistant (Yamazaki et al., 2022; Chua et al., 2019; Mason et al., 2024). This inter-individual variability highlights the need to better understand the neural changes associated with vulnerability to sleep loss (Mason et al., 2024; Subramaniyan et al., 2024).

Electroencephalography (EEG) has been widely used to investigate the neural effects of sleep deprivation because of its high temporal resolution and ability to characterize neural oscillatory activity (Cohen, 2017). Previous studies have reported sleep deprivation-related changes across several frequency bands, particularly in the theta, alpha, and beta ranges (Hudson et al., 2020; Harel et al., 2025). Increased theta activity has frequently been associated with greater sleep pressure and reduced alertness, whereas changes in alpha- and beta-band activity may reflect altered arousal regulation, cortical activation, and cognitive control (Snipes et al., 2022; Andrillon et al., 2021; Krause et al., 2017). Many studies have characterized these effects at the group level using conventional spectral power measures (Hudson et al., 2020; Harel et al., 2025). Although mean oscillatory power provides a useful summary of the average level of oscillatory activity over a recording period, it does not directly characterize the temporal stability of that activity within the recording. Thus, conventional mean power analyses leave open the question of whether sleep deprivation alters not only the average level of band-limited oscillatory activity, but also the magnitude of its fluctuations across consecutive epochs. Such fluctuations may be behaviorally relevant because transient sleep-like neural activity during wakefulness has been linked to attentional lapses and slowed responses (Andrillon et al., 2021; Yang et al., 2025; Pinggal et al., 2026). Measures of oscillatory variability may therefore complement mean spectral power by characterizing across-epoch fluctuations in oscillatory activity (Angulo-Ruiz et al., 2021). Nevertheless, whether sleep deprivation-related changes in oscillatory variability are associated with individual differences in vigilance decline remains unclear. More broadly, EEG temporal dynamics and neural variability have been studied using several approaches, including EEG microstate analysis, entropy- or complexity-based measures, and epoch-level variability metrics such as the coefficient of variation (Haydock et al., 2025; Lau et al., 2022; Angulo-Ruiz et al., 2021). These approaches have shown that EEG activity contains temporally varying information that may not be fully captured by time-averaged spectral power alone. The present study builds on this broader literature by focusing specifically on the across-epoch variability of band-limited relative power during resting-state EEG.

To address this question, the present study examined mean relative power and power variability across consecutive resting-state EEG epochs in the theta, alpha, and beta bands under normal sleep and sleep deprivation conditions. Primary analyses were conducted at the regional level, and complementary channel-level analyses were used to further characterize the spatial distribution of sleep deprivation-related EEG oscillatory changes. We then examined whether changes in these oscillatory features were associated with individual differences in vigilance decline, indexed by changes in PVT median reaction time. This approach allowed mean-level and variability-based measures to be evaluated within the same analytical framework and their relationships with individual vulnerability to sleep loss to be compared. As an exploratory extension, potential sex-related differences in neural oscillation changes and PVT performance changes, as well as the associations between these changes, were also examined.

## 2 Materials and methods

### 2.1 Dataset and participants

We obtained data from the publicly available OpenNeuro dataset *A Resting-state EEG Dataset for Sleep Deprivation* (accession number: ds004902; version 1.0.4) (Xiang et al., 2024), which was originally described by (Xiang et al. 2024). The dataset includes resting-state EEG recordings from 71 participants under two conditions: normal sleep (NS; Session 1) and sleep deprivation (SD; Session 2). In the released dataset, NS recordings were labeled as Session 1 and SD recordings as Session 2. These labels denote experimental conditions rather than the chronological order of data acquisition; the acquisition order was counterbalanced across participants and is provided in the metadata. The present study focused on resting-state EEG recordings and PVT-derived behavioral measures obtained under both conditions. The EEG data analyzed here were not acquired during PVT performance. Although the original dataset included both eyes-open and eyes-closed resting-state EEG recordings, only the eyes-open recordings were included in the present analyses. During these recordings, participants were instructed to remain awake with their eyes open.

Participant subsets differed according to the availability of valid EEG and PVT data (Table 1). A total of 64 participants had usable resting-state EEG recordings under both NS and SD conditions and were included in analyses of EEG oscillatory changes and sex-related oscillatory differences; this sample comprised 33 males and 31 females. Among these 64 participants, 28 had valid PVT data under both conditions. This subgroup comprised 18 males and 10 females and was used for EEG–PVT association analyses and analyses of sex-related differences in PVT performance.

**Table 1:** Sample information and data availability.

Analysis sample	Sample size	Male/female	Data included
EEG analysis	64	33/31	EEG under NS and SD
PVT analysis	28	18/10	PVT under NS and SD
EEG–PVT association analysis	28	18/10	EEG and PVT under NS and SD

NS, normal sleep; SD, sleep deprivation; PVT, psychomotor vigilance task.

### 2.2 EEG data pre-processing

EEG data were pre-processed in MNE-Python (Gramfort et al., 2013, 2014). We imported the raw EEGLAB recordings, applied the standard 10–20 montage, downsampled the data to 500 Hz, and used a 1–30 Hz band-pass filter. Continuous recordings were visually inspected, and bad channels were manually marked based on persistent high-amplitude noise, abrupt non-physiological fluctuations, or abnormal periodic activity inconsistent with neighboring electrodes. The same criteria were applied to both conditions. Marked channels were excluded from average re-referencing and ICA fitting. ICA was performed using the extended Infomax algorithm, retaining components explaining 99% of the variance (Lee et al., 1999; Jung et al., 2000). Ocular components were identified using Fp1 and Fp2 as proxy EOG channels and removed when the absolute EOG score exceeded 0.85. Muscle-related components were identified using ICLabel (Pion-Tonachini et al., 2019) and removed when the muscle-artifact probability exceeded 0.85. The marked bad channels were then interpolated, and the cleaned data were divided into 75 non-overlapping 4-s epochs, with epochs exceeding 200 μV peak-to-peak rejected.

Although pre-processing was not formally blinded to session labels, decisions were based only on EEG signal quality and were made without reference to PVT performance or subsequent EEG–behavior results. The mean numbers of marked bad channels were 0.39 ± 1.58 and 0.17 ± 0.52 for normal sleep and sleep deprivation, respectively; the corresponding numbers of removed ICA components were 2.64 ± 1.77 and 3.11 ± 2.44, and the numbers of rejected epochs were 5.17 ± 9.56 and 3.98 ± 5.25 out of 75. These descriptive quality-control summaries did not suggest greater overall pre-processing-related data loss in the sleep deprivation condition.

### 2.3 Oscillatory feature extraction

The 4-s epoch length was selected as a compromise between providing sufficient frequency resolution for the pre-defined frequency bands and retaining enough epochs to estimate across-epoch variability reliably. It also preserves sensitivity to relatively short-term fluctuations in oscillatory activity while limiting the temporal averaging of transient changes. For each cleaned 4-s epoch and each EEG channel, power spectral density (PSD) was estimated using Welch's method (Welch, 1967). Band-specific power was calculated by integrating the PSD within the theta (4–8 Hz), alpha (8–13 Hz), and beta (13–30 Hz) frequency bands. These band boundaries are consistent with conventional EEG spectral definitions and have also been used for theta, alpha, and beta bands in previous sleep-deprivation EEG studies (Xiang et al., 2024; Lian et al., 2023). Total power within the 4–30 Hz range was used as the denominator for relative power calculation. For each epoch, channel, and frequency band, epoch-wise relative power was defined using Equation (1):


$$\begin{array}{l} RP_{e}\left(B\right)=\frac{\int_{B}P_{e}\left(f\right)\text{d}f}{\int_{F}P_{e}\left(f\right)\text{d}f} \end{array}$$ (1)

where *P*_*e*_(*f*) denotes the PSD at frequency *f* for epoch *e*, *B* denotes the frequency band of interest, and F denotes the total frequency range of 4–30 Hz.

For each subject, session, channel, and frequency band, mean relative power was calculated using Equation (2) by averaging the epoch-wise relative power values across epochs:


$$\begin{array}{l} RP\left(B\right)={\mathrm{mean}}_{e}\left[RP_{e}\left(B\right)\right] \end{array}$$ (2)

Power variability was quantified using Equation (3) as the standard deviation of the epoch-wise relative power values across epochs:


$$\begin{array}{l} PV\left(B\right)={\mathrm{SD}}_{e}\left[RP_{e}\left(B\right)\right] \end{array}$$ (3)

where mean_*e*_ and SD_*e*_ denote the mean and standard deviation across epochs, respectively.

**Figure 1:**
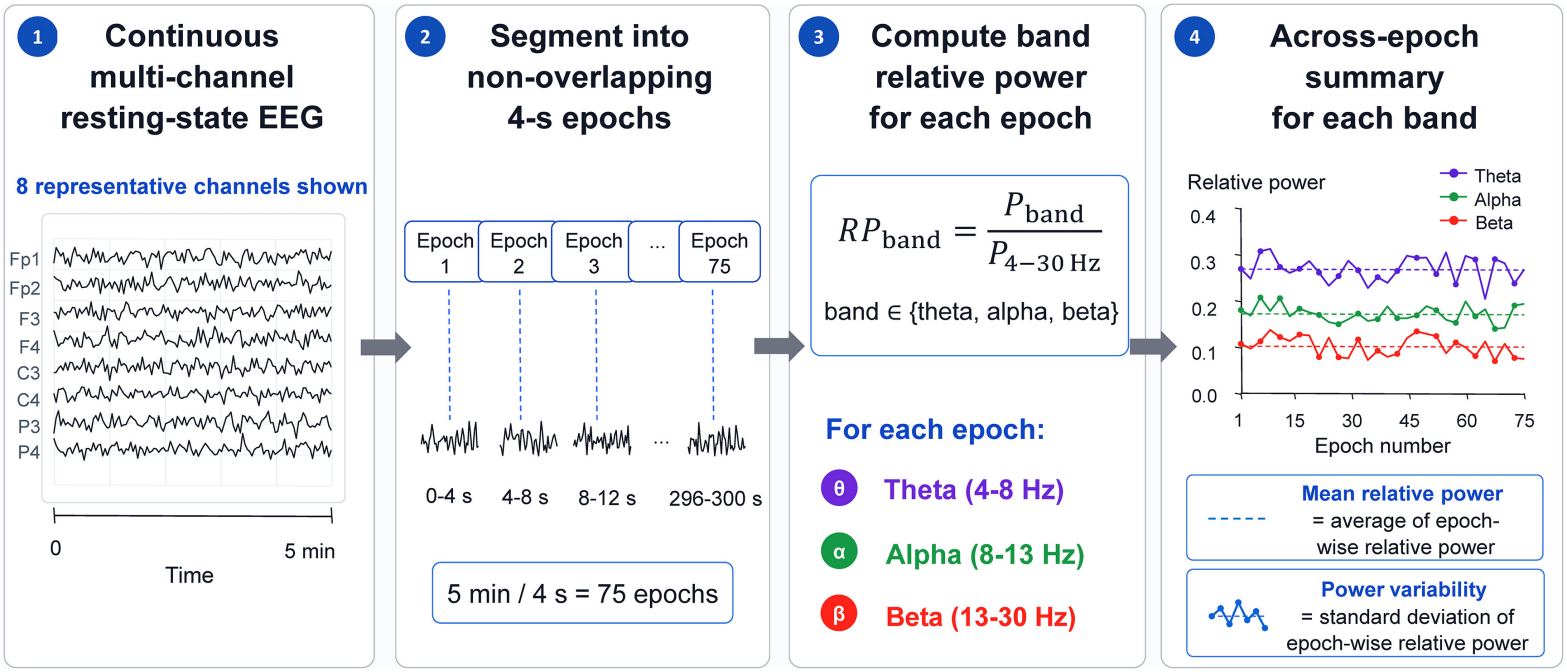
Schematic illustration of oscillatory feature extraction. The cleaned continuous multi-channel resting-state EEG recording was segmented into 75 non-overlapping 4-s epochs. For each epoch and EEG channel, relative power was calculated within the theta, alpha, and beta bands using total power within 4–30 Hz as the denominator. Mean relative power and power variability were subsequently calculated across epochs as the mean and standard deviation of the epoch-wise relative power values, respectively.

The computation of mean relative power and power variability from epoch-wise relative power values is illustrated schematically in Figure 1.

Aperiodic-adjusted oscillatory features were additionally computed to further evaluate whether the primary findings remained robust after accounting for the aperiodic component. The power spectral density of each 4-s epoch and EEG channel was parameterized using FOOOF (version 1.1) (Donoghue et al., 2020). Power spectral density was estimated using Welch's method over 1–30 Hz, and spectral parameterization was performed over 2–30 Hz using a fixed aperiodic mode, with peak-width limits of 1–8 Hz, a maximum of six peaks, a minimum peak height of 0.05, and a peak-detection threshold of 2.0. For each epoch and channel, the fitted aperiodic component was subtracted from the log-transformed power spectrum to obtain a residual spectrum. Residual spectral values were averaged within the theta, alpha, and beta bands. The mean and standard deviation of the resulting epoch-wise band values were then calculated and are referred to as residual power and residual variability, respectively. Channel-level values were subsequently averaged within the three predefined ROIs.

### 2.4 Behavioral measures

Behavioral performance under the NS and SD conditions was assessed using the psychomotor vigilance task (PVT), which is widely used to measure alertness and response speed in sleep-loss research (Basner et al., 2021; Hudson et al., 2020). Responses faster than 100 ms were excluded as false starts. To reduce the influence of extremely delayed responses, reaction times longer than 2,000 ms were also excluded before calculating median reaction time. Median reaction time was used as the primary behavioral indicator because it is a commonly reported PVT outcome and provides a robust summary of response latency (Basner and Dinges, 2011). Vigilance change was quantified as the difference in median reaction time between the SD and NS conditions (SD minus NS), with larger positive values indicating greater reaction-time slowing after sleep deprivation.

### 2.5 Scalp region grouping

**Figure 2:**
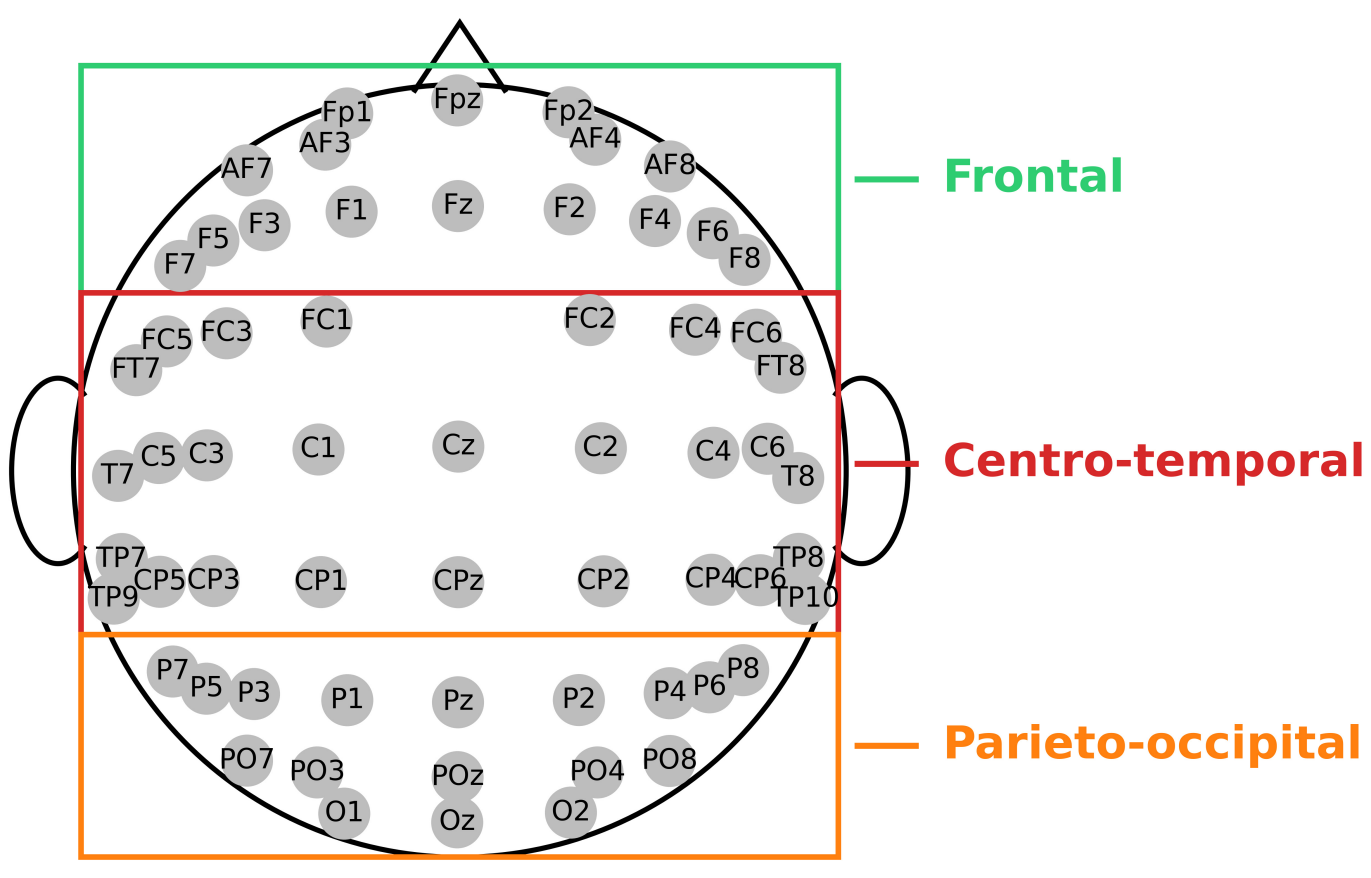
Scalp regions of interest for ROI-level analyses. Green, red, and orange indicate the frontal, centro-temporal, and parieto-occipital regions, respectively.

For region-level analyses, electrode nomenclature and locations followed the standard international 10–10 system (Acharya et al., 2016). Based on the regional framework used by Quiquempoix et al. (2022), we grouped the EEG channels into three broad scalp regions of interest (ROIs): frontal, centro-temporal, and parieto-occipital, as illustrated in Figure 2. The frontal region included Fp1, Fpz, Fp2, AF7, AF3, AF4, AF8, F7, F5, F3, F1, Fz, F2, F4, F6, and F8. The centro-temporal region included FT7, FC5, FC3, FC1, FC2, FC4, FC6, FT8, T7, C5, C3, C1, Cz, C2, C4, C6, T8, TP7, CP5, CP3, CP1, CPz, CP2, CP4, CP6, TP8, TP9, and TP10. The parieto-occipital region included P7, P5, P3, P1, Pz, P2, P4, P6, P8, PO7, PO3, POz, PO4, PO8, O1, Oz, and O2. For each ROI, we averaged the channel-wise measures to obtain region-level features.

### 2.6 Statistical analysis

We compared ROI-level oscillatory features between the NS and SD conditions using two-sided paired permutation tests, with the mean within-subject difference as the test statistic (Winkler et al., 2014). The analyses were organized into two predefined feature families: relative band power and the across-epoch variability of relative band power. Within each family, we applied the Benjamini–Hochberg false discovery rate (FDR) procedure across the nine ROI-level comparisons comprising three frequency bands and three scalp regions (Benjamini and Hochberg, 1995). We also generated group-averaged SD–NS topographic maps for the six EEG metrics to visualize the scalp distribution of EEG changes. At each electrode, we applied one-sample t-tests against zero to the SD–NS change scores. For each feature family, p-values were corrected across the 183 channel-level comparisons comprising three frequency bands and 61 electrodes using the same FDR procedure.

To assess whether EEG changes were associated with individual differences in vigilance decline, we calculated Spearman correlations between ROI-level SD–NS EEG changes and SD–NS changes in PVT median reaction time (Gibbings et al., 2021; Zhang et al., 2025; Tramonti Fantozzi et al., 2022). Larger positive PVT changes indicated greater vigilance decline. We conducted the analyses separately for the relative power and power variability families and applied the Benjamini–Hochberg false discovery rate (FDR) procedure across the nine ROI-level comparisons comprising three frequency bands and three scalp regions within each family (Benjamini and Hochberg, 1995). As a targeted follow-up control analysis, we examined whether the observed theta power variability associations were separable from mean theta relative power. Partial Spearman correlations were performed for the frontal and centro-temporal ROIs, where ROI-level theta variability associations were observed in the main analysis. The corresponding change in mean theta relative power was included as the control variable. For the significant theta power variability–PVT associations, we repeated the correlation analysis after excluding one participant at a time to determine whether any single participant drove the observed associations. A targeted baseline analysis was conducted in the frontal and centro-temporal ROIs to examine whether theta power variability under normal sleep was associated with PVT median reaction time under normal sleep. This analysis was performed to determine whether the change-score associations reflected similar baseline relationships under the normal sleep condition. We also examined the scalp distribution of EEG–PVT associations by calculating channel-wise Spearman correlations between SD–NS EEG changes and PVT changes for all six EEG metrics. Within each feature family, p-values were corrected across the 183 channel-level comparisons comprising three frequency bands and 61 electrodes. We visualized the channel-wise correlation coefficients as scalp topographic maps.

For the aperiodic-adjusted analysis, sleep-condition differences in residual power and residual variability were examined using the same two-sided paired permutation tests and effect-size calculations as in the primary analyses. Associations between changes in these features and changes in PVT median reaction time were assessed using Spearman correlations. FDR correction was applied separately within the residual-power and residual-variability families across the nine band-by-ROI comparisons, separately for the sleep-condition comparisons and EEG–PVT association analyses.

To assess the robustness of the findings to the selected epoch length, mean relative power and power variability were recomputed using non-overlapping epochs of 2, 8, and 10 s, in addition to the primary 4-s epochs. For each epoch length, the same frequency-band definitions, ROI definitions, and feature-calculation procedures were applied. The sleep-condition comparisons and EEG–PVT association analyses were then repeated using the same statistical procedures as in the primary analyses. FDR correction was applied separately within the relative-power and relative-power-variability families across the nine band-by-ROI comparisons for each epoch length, and separately for the sleep-condition comparisons and EEG–PVT association analyses.

Exploratory sex-related analyses assessed differences in sleep deprivation responses between male and female participants (Ołpińska-Lischka et al., 2020; Hajali et al., 2019; Santhi et al., 2016). ROI-level SD–NS oscillatory changes were compared between sexes using Welch's t-tests. Relative power and power variability were analyzed separately, with FDR correction across the nine comparisons within each feature family. Sex differences in SD–NS changes in PVT median reaction time were assessed using a median-difference permutation test, with Cliff's delta reported as the effect size (Cliff, 2014). We examined the two FDR-significant theta power variability–PVT associations separately in males and females, yielding four Spearman correlations, and adjusted the corresponding p-values across these four tests. Sex-label permutation tests compared correlation strength between males and females, whereas linear regression models with an interaction between sex and the change in theta power variability assessed differences in EEG–PVT slopes. Each between-sex analysis used FDR correction across the two ROIs.

## 3 Results

### 3.1 Changes in EEG oscillatory features after sleep deprivation

**Figure 3:**
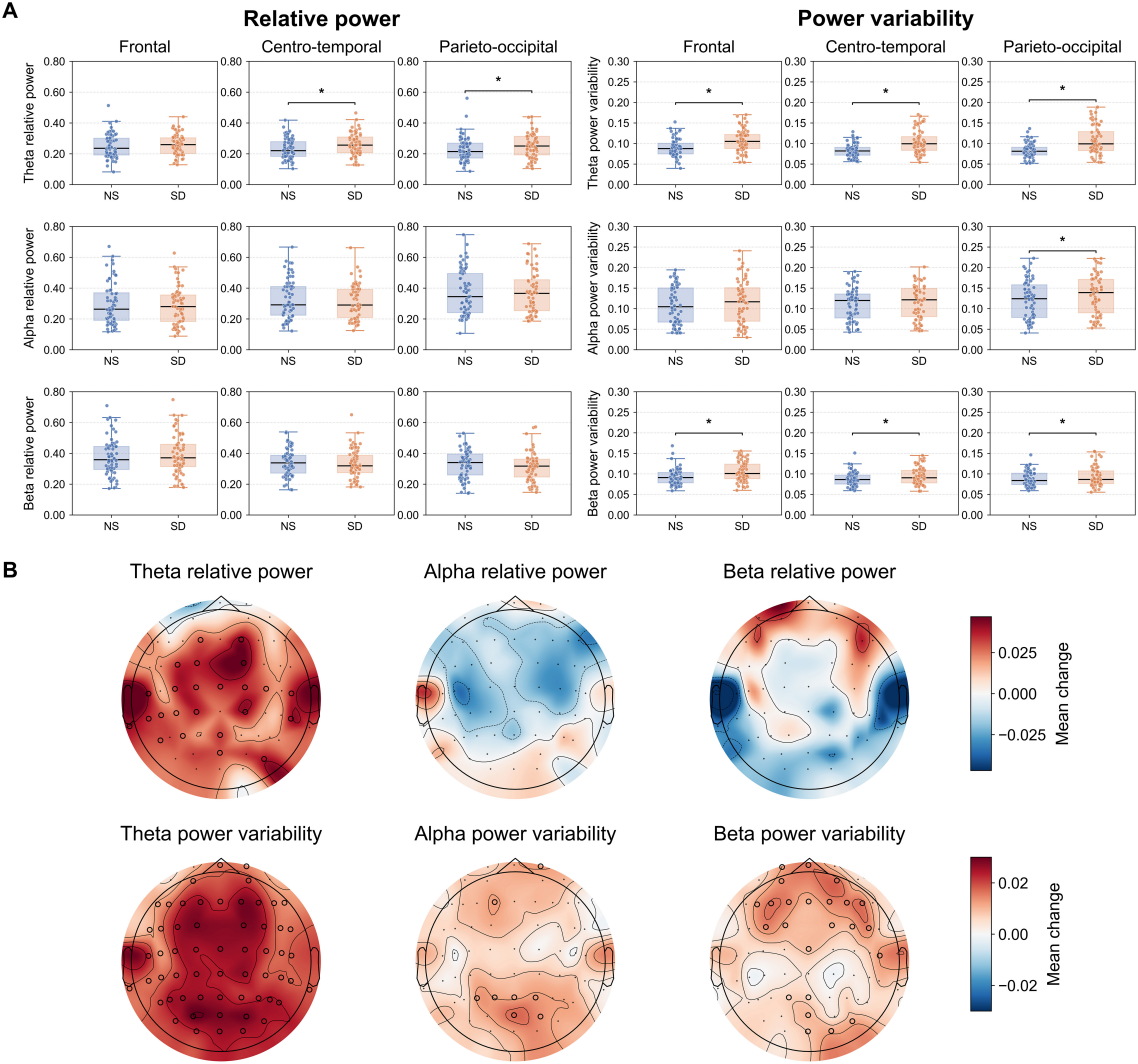
Changes in EEG oscillatory features after sleep deprivation. **(A)** ROI-level distributions of relative power and power variability under normal sleep (NS) and sleep deprivation (SD) conditions across the frontal, centro-temporal, and parieto-occipital regions. Asterisks indicate comparisons between NS and SD that remained significant after FDR correction. Full statistical results, including FDR-adjusted *p* values and effect sizes, are provided in Table 2. **(B)** Channel-level topographic maps showing SD–NS changes in relative power and power variability in the theta, alpha, and beta bands. Warm colors indicate increases after sleep deprivation, whereas cool colors indicate decreases. Marked electrodes indicate channels surviving within-family FDR correction.

Sleep deprivation was associated with changes in neural oscillatory features across frequency bands and scalp regions. As shown in Figure 3A, ROI-level analyses revealed that SD-related changes were more prominent and consistent for power variability than for relative power.

Within the relative power family, theta relative power showed a slight upward shift after SD, particularly in the centro-temporal and parieto-occipital regions. Paired permutation tests confirmed significant increases in theta relative power in the centro-temporal and parieto-occipital regions after FDR correction, whereas the frontal region did not survive correction. Alpha and beta relative power showed no reliable ROI-level changes across the three regions.

Power variability showed broader and more robust SD-related changes. Theta power variability was higher in SD than in NS across all three ROIs, with significant increases in the frontal, centro-temporal, and parieto-occipital regions after FDR correction. Alpha power variability showed a significant increase only in the parieto-occipital region, whereas the frontal increase did not survive FDR correction and the centro-temporal region showed no significant change. Beta power variability increased significantly across all three ROIs, with the largest effect observed in the frontal region; however, the magnitude of these increases was relatively modest compared with theta power variability. Detailed ROI-level statistical results are provided in Table 2.

**Table 2:** ROI-level comparisons of oscillatory features between normal sleep and sleep deprivation.

Feature family	Band	ROI	Mean change (SD–NS)	Cohen's *d*_*z*_	Uncorrected *p*	FDR-adjusted *p*
Relative power	Theta	Frontal	0.0114	0.149	0.237	0.329
		Centro-temporal	0.0297	0.536	< 0.001	0.002
		Parieto-occipital	0.0259	0.354	0.005	0.023
	Alpha	Frontal	−0.0121	−0.140	0.256	0.329
		Centro-temporal	−0.0153	−0.212	0.094	0.248
		Parieto-occipital	−0.0002	−0.003	0.981	0.981
	Beta	Frontal	0.0193	0.196	0.120	0.248
		Centro-temporal	−0.0070	−0.104	0.412	0.464
		Parieto-occipital	−0.0134	−0.187	0.138	0.248
Power variability	Theta	Frontal	0.0177	0.565	< 0.001	< 0.001
		Centro-temporal	0.0206	0.767	< 0.001	< 0.001
		Parieto-occipital	0.0251	0.783	< 0.001	< 0.001
	Alpha	Frontal	0.0088	0.245	0.051	0.057
		Centro-temporal	0.0051	0.173	0.171	0.171
		Parieto-occipital	0.0112	0.306	0.016	0.021
	Beta	Frontal	0.0125	0.480	< 0.001	0.002
		Centro-temporal	0.0063	0.325	0.012	0.018
		Parieto-occipital	0.0059	0.319	0.011	0.018

Mean change was calculated as SD–NS. Comparisons were performed using two-sided paired permutation tests. FDR correction was applied separately within each feature family. All analyses included 64 participants.

At the channel level, topographic maps showed the spatial distribution of SD–NS changes across the scalp (Figure 3B). For relative power, significant increases were confined mainly to the theta band and were concentrated in the centro-temporal region, with additional effects at several frontal and parieto-occipital electrodes. No channels survived FDR correction for alpha or beta relative power. Theta power variability showed the most widespread increase, with 58 of 61 channels surviving FDR correction. Significant increases in beta power variability were observed mainly over frontal and fronto-central areas, with additional posterior effects. Alpha power variability showed a more restricted pattern, involving frontal and parieto-occipital electrodes. The numbers and regional distributions of significant channels are summarized in Table 3. Overall, theta power variability showed the broadest channel-level changes, whereas the alpha- and beta-band effects were more spatially restricted.

**Table 3:** Summary of channel-level oscillatory changes surviving FDR correction following sleep deprivation.

Feature family	Band	Direction	Overall (*n*/61)	Frontal (*n*/16)	Centro-temporal (*n*/28)	Parieto-occipital (*n*/17)
Relative power	Theta	Increase	24/61	2/16	17/28	5/17
	Alpha	—	0/61	0/16	0/28	0/17
	Beta	—	0/61	0/16	0/28	0/17
Power variability	Theta	Increase	58/61	13/16	28/28	17/17
	Alpha	Increase	8/61	2/16	0/28	6/17
	Beta	Increase	24/61	11/16	7/28	6/17

Counts indicate channels surviving within-family FDR correction across three frequency bands and 61 channels. All significant SD–NS changes were positive. Two-sided one-sample *t*-tests against zero were applied to the SD–NS change scores from 64 participants.

### 3.2 Associations between oscillatory changes and vigilance decline

**Figure 4:**
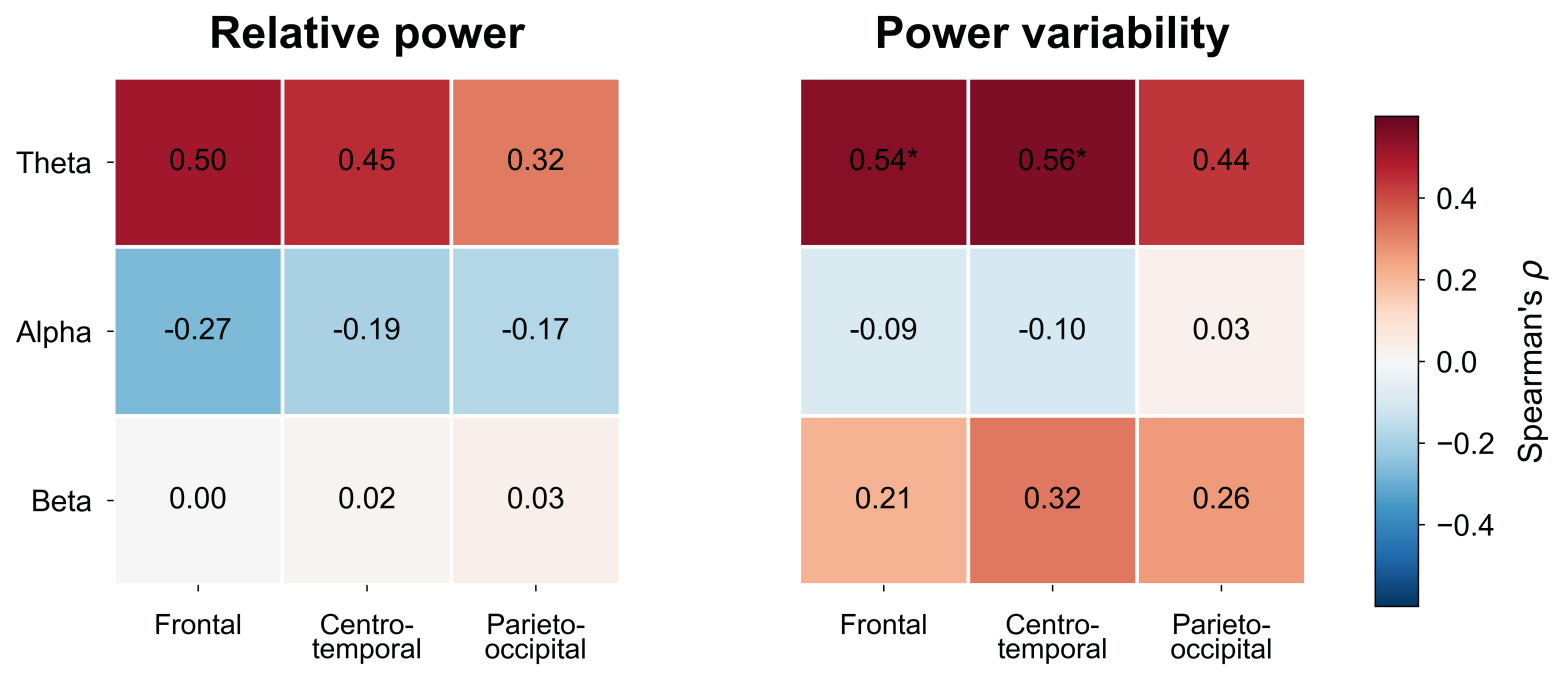
ROI-level associations between sleep deprivation-related oscillatory changes and vigilance decline. Heatmaps show Spearman correlations between SD–NS oscillatory changes and SD–NS changes in PVT median reaction time. Asterisks indicate correlations surviving FDR correction.

We examined associations between sleep deprivation-related oscillatory changes and individual differences in vigilance decline using Spearman correlations between ROI-level SD–NS oscillatory changes and SD–NS changes in PVT median reaction time. The statistical results are reported in Table 4, and Figure 4 shows the corresponding correlation patterns across oscillatory features and ROIs.

**Table 4:** ROI-level correlations between oscillatory changes and vigilance decline.

Feature family	Band	ROI	Spearman's ρ	Uncorrected *p*	FDR-adjusted *p*
Relative power	Theta	Frontal	0.504	0.006	0.056
		Centro-temporal	0.451	0.016	0.072
		Parieto-occipital	0.316	0.101	0.303
	Alpha	Frontal	–0.268	0.168	0.379
		Centro-temporal	–0.193	0.324	0.566
		Parieto-occipital	–0.174	0.377	0.566
	Beta	Frontal	0.000	1.000	1.000
		Centro-temporal	0.016	0.935	1.000
		Parieto-occipital	0.031	0.877	1.000
Power variability	Theta	Frontal	0.544	0.003	**0.012**
		Centro-temporal	0.558	0.002	**0.012**
		Parieto-occipital	0.438	0.020	0.060
	Alpha	Frontal	–0.093	0.638	0.717
		Centro-temporal	–0.102	0.604	0.717
		Parieto-occipital	0.026	0.894	0.894
	Beta	Frontal	0.214	0.273	0.410
		Centro-temporal	0.320	0.097	0.219
		Parieto-occipital	0.262	0.178	0.321

Spearman correlations were calculated between SD–NS changes in oscillatory features and SD–NS changes in PVT median reaction time. Positive correlations indicate that larger increases in oscillatory features were associated with greater PVT slowing. FDR-adjusted *p* values were calculated separately within the power and variability feature families. Bold values indicate FDR-adjusted *p* < 0.05. Analyses were conducted in the EEG–PVT subset (*n* = 28).

Theta-band features showed the strongest associations with PVT slowing. Theta relative power was positively correlated with PVT median reaction time changes across all three ROIs, although none of these associations survived within-family FDR correction. Alpha relative power showed weak negative correlations, whereas beta relative power showed little association with PVT changes.

Theta power variability was most strongly and consistently associated with vigilance decline. The correlations in the frontal ROI (ρ = 0.54, uncorrected *p* = 0.003, FDR-adjusted *p* = 0.012) and centro-temporal ROI (ρ = 0.56, uncorrected *p* = 0.002, FDR-adjusted *p* = 0.012) survived FDR correction. A positive correlation was also observed in the parieto-occipital ROI, but it did not survive correction. Alpha power variability showed only weak correlations with PVT changes. Beta power variability was positively correlated with PVT changes, particularly in the centro-temporal region, but none of these associations survived FDR correction.

**Figure 5:**
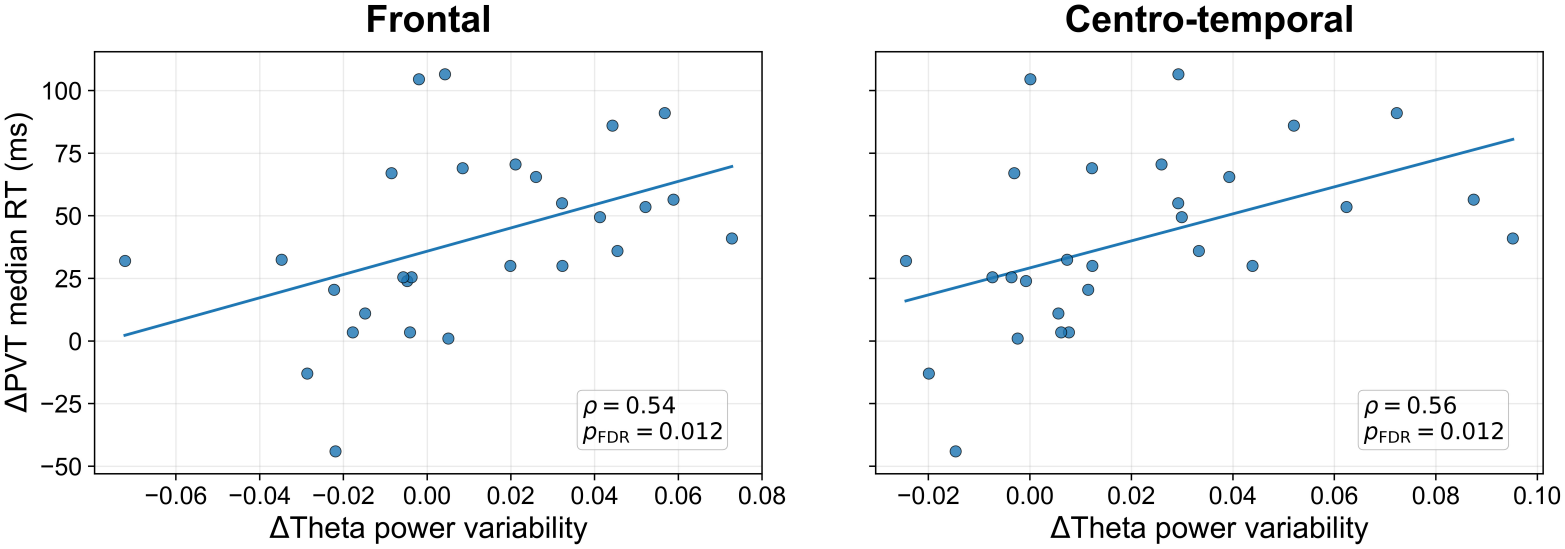
Associations between changes in theta power variability and vigilance decline. Scatter plots show the associations between SD–NS changes in theta power variability and SD–NS changes in PVT median reaction time in the frontal and centro-temporal ROIs. Solid lines indicate ordinary least-squares linear fits for visualization. Reported statistics are Spearman's ρ and FDR-adjusted *p* values.

To illustrate the significant associations, scatter plots were generated for theta power variability in the frontal and centro-temporal ROIs, as shown in Figure 5. In both ROIs, larger SD–NS increases in theta power variability were associated with larger increases in PVT median reaction time, indicating greater vigilance decline.

To further examine whether these variability-behavior associations could be explained by changes in mean theta relative power, we performed a targeted partial Spearman correlation analysis in the two ROIs that showed significant associations in the main analysis. After controlling for the corresponding change in mean theta relative power, the association between theta power variability and PVT slowing was attenuated but remained positive in both ROIs. The partial association was weaker in the frontal ROI (partial ρ = 0.28, *p* = 0.149), whereas a moderate association remained in the centro-temporal ROI (partial ρ = 0.39, *p* = 0.039). These results suggest that theta power variability shares variance with mean theta relative power but is not fully reducible to it, particularly in the centro-temporal region.

We assessed whether the key ROI-level associations were strongly influenced by any single participant by repeating the Spearman correlation analysis after excluding one participant at a time. Across all iterations, the correlations remained positive in both the frontal region, ρ ∈ [0.508, 0.594], and the centro-temporal region, ρ ∈ [0.515, 0.636]. Neither association appeared to be driven by a single participant.

Taken together, these ROI-level analyses indicate that theta power variability showed the most prominent associations with individual differences in vigilance decline among the examined oscillatory features.

We further examined whether the relationship between theta power variability and PVT performance was also present under the normal sleep condition. Under normal sleep, theta power variability was not associated with PVT median reaction time in either the frontal ROI (ρ = 0.074, *p* = 0.708) or the centro-temporal ROI (ρ = 0.114, *p* = 0.563). Thus, the change-score associations were not mirrored by comparable baseline associations under normal sleep, suggesting that the observed relationship was more evident for sleep deprivation-related changes than for baseline individual differences in vigilance.

**Figure 6:**
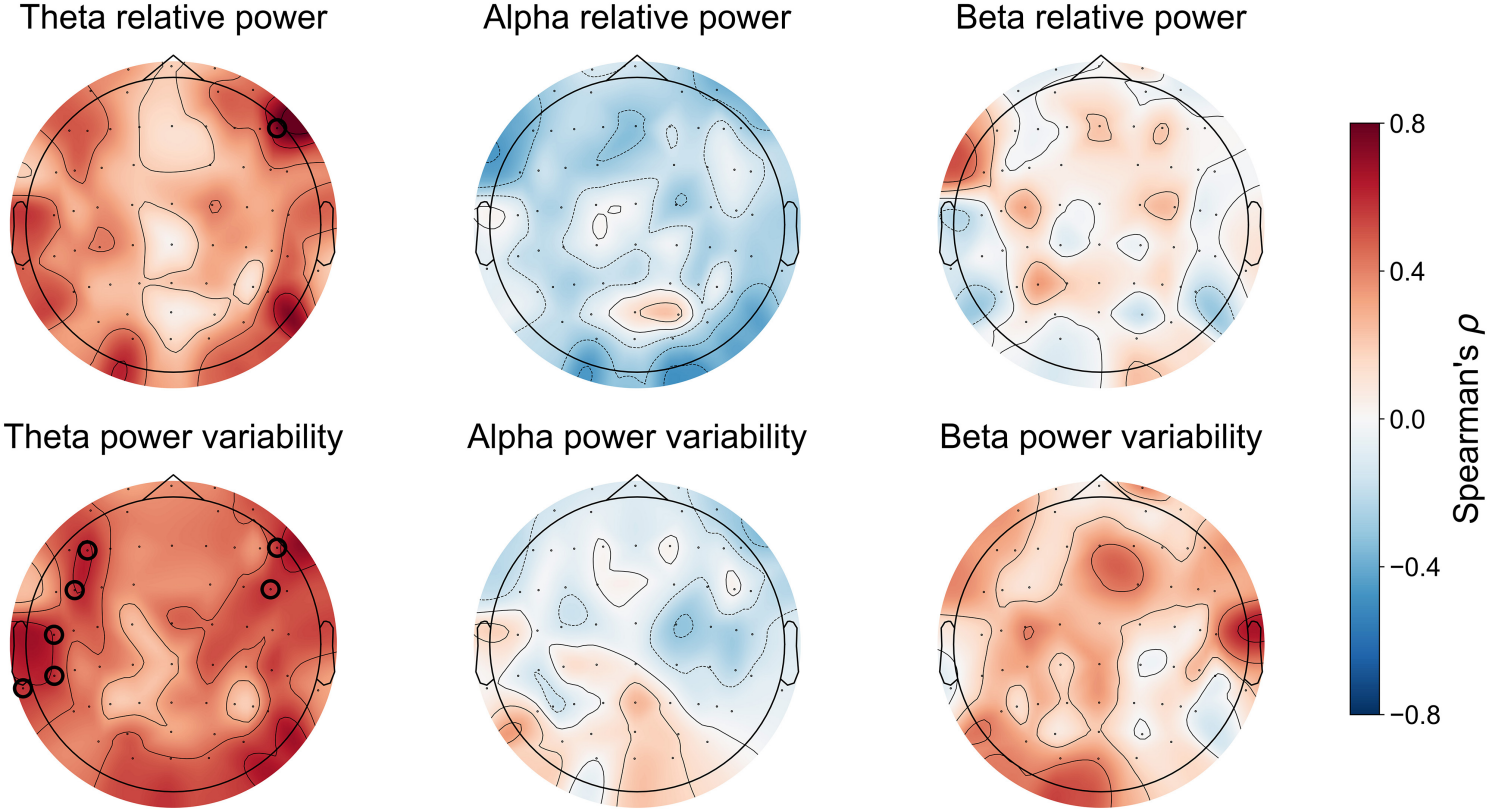
Topographical distribution of EEG–PVT correlations. Topographic maps show channel-wise Spearman correlations between SD–NS EEG changes and SD–NS changes in PVT median reaction time. Marked electrodes indicate channels surviving within-family FDR correction.

Channel-level Spearman correlation analyses were used to examine the scalp distribution of EEG–PVT associations (Figure 6). Theta-band features showed the clearest spatial patterns. Theta power variability and theta relative power were positively associated with PVT slowing across much of the scalp. For theta power variability, all 61 electrodes showed positive ρ values, and seven electrodes survived within-family FDR correction: F5, F8, FC5, FC6, T7, TP7, and TP9. Theta relative power also showed broadly positive correlations, although only F8 survived FDR correction. Detailed statistics for channels surviving FDR correction are provided in Table 5.

**Table 5:** Channel-level EEG–PVT associations surviving FDR correction.

EEG oscillatory feature	Channel	Spearman's ρ	Uncorrected *p*	FDR-adjusted *p*
Theta relative power	F8	0.730	< 0.001	0.002
Theta power variability	F5	0.621	< 0.001	0.042
	FC5	0.599	< 0.001	0.042
	T7	0.589	< 0.001	0.042
	TP9	0.582	0.001	0.042
	FC6	0.581	0.001	0.042
	TP7	0.574	0.001	0.042
	F8	0.565	0.002	0.046

Only channels surviving within-family FDR correction are shown. EEG and PVT changes were calculated as SD–NS. Positive correlations indicate that greater EEG increases were associated with greater slowing in PVT median reaction time. FDR correction was applied separately within the relative power and power variability families across three frequency bands and 61 channels. Analyses included 28 participants.

In contrast, alpha relative power showed a mostly weak negative pattern, whereas beta relative power showed weak and spatially mixed correlations. Alpha power variability showed low correlations with PVT changes, and beta power variability showed a mild positive tendency across many electrodes, but no electrodes survived FDR correction.

These channel-level results extended the ROI-level findings by providing finer spatial localization of the theta power variability–PVT associations, with significant effects mainly distributed over frontal, fronto-central, and temporal electrodes.

### 3.3 Robustness analyses of oscillatory features

#### 3.3.1 Aperiodic-adjusted analysis

After removal of the fitted aperiodic background, theta-band residual power remained significantly higher under sleep deprivation than under normal sleep in all three ROIs, whereas alpha-band residual power showed no significant condition differences. Beta-band residual power decreased in the centro-temporal and parieto-occipital ROIs. Residual variability increased significantly after sleep deprivation across all frequency bands and ROIs after FDR correction (Table 6).

**Table 6:** Aperiodic-adjusted analyses of oscillatory features.

Feature family	Band	ROI	Mean change (SD–NS)	Cohen's *d*_*z*_	FDR-adjusted *p* for comparison	Spearman's ρ with PVT slowing	FDR-adjusted *p* for association
Residual power	Theta	F	0.0357	0.567	< 0.001	0.226	0.433
		CT	0.0470	0.826	< 0.001	0.191	0.433
		PO	0.0484	0.703	< 0.001	0.195	0.433
	Alpha	F	−0.0167	−0.115	0.533	−0.299	0.433
		CT	−0.0099	−0.080	0.586	−0.214	0.433
		PO	0.0119	0.088	0.586	−0.188	0.433
	Beta	F	−0.0011	−0.040	0.752	0.293	0.433
		CT	−0.0058	−0.293	0.037	0.128	0.517
		PO	−0.0101	−0.456	< 0.001	0.161	0.464
Residual variability	Theta	F	0.0098	0.486	0.001	0.442	0.083
		CT	0.0093	0.553	< 0.001	0.598	0.007
		PO	0.0105	0.523	< 0.001	0.299	0.275
	Alpha	F	0.0151	0.417	0.003	−0.074	0.798
		CT	0.0085	0.266	0.034	−0.084	0.798
		PO	0.0156	0.352	0.009	0.245	0.375
	Beta	F	0.0028	0.391	0.004	0.220	0.390
		CT	0.0027	0.435	0.002	0.404	0.100
		PO	0.0027	0.348	0.009	−0.002	0.992

Mean change was calculated as SD minus NS. Group-level comparisons were performed using two-sided paired permutation tests in 64 participants. Associations with PVT median reaction time slowing were assessed using Spearman correlations in 28 participants. FDR correction was applied separately within the residual-power and residual-variability families across nine band-by-ROI comparisons for the group comparisons and EEG–PVT associations. F, frontal; CT, centro-temporal; PO, parieto-occipital.

In the EEG–PVT analyses, none of the associations involving residual power survived FDR correction. For residual variability, only the theta-band association in the centro-temporal ROI remained significant after correction (ρ = 0.598, FDR-adjusted *p* = 0.007). The corresponding frontal association remained positive but did not survive correction (ρ = 0.442, FDR-adjusted *p* = 0.083), and no other association was significant.

Overall, the widespread sleep deprivation-related increase in theta-band residual variability remained evident after aperiodic adjustment, suggesting that the group-level effect was not fully explained by the fitted aperiodic component. Compared with the primary analysis, however, the EEG–PVT association became more spatially restricted, with only the centro-temporal theta-band residual variability association remaining significant after FDR correction.

#### 3.3.2 Epoch-length sensitivity analysis

The epoch-length sensitivity analysis was conducted for both feature families across all three frequency bands and all three ROIs. Across the full set of analyses, sleep deprivation-related increases in theta power variability were consistent across the examined epoch lengths, with significant increases observed in all three ROIs after FDR correction for the 2-, 4-, 8-, and 10-s epochs. Because the primary EEG–PVT associations were observed for theta power variability in the frontal and centro-temporal ROIs, Table 7 summarizes the key results for these two regions.

**Table 7:** Epoch-length sensitivity analyses of theta power variability.

Epoch length	ROI	Mean change (SD–NS)	Cohen's *d*_*z*_	FDR-adjusted *p* for comparison	Spearman's ρ with PVT slowing	FDR-adjusted *p* for association
2 s	F	0.0153	0.476	0.001	0.515	0.023
	CT	0.0195	0.712	< 0.001	0.532	0.023
4 s	F	0.0177	0.566	< 0.001	0.544	0.012
	CT	0.0206	0.768	< 0.001	0.558	0.012
8 s	F	0.0168	0.529	0.001	0.515	0.023
	CT	0.0195	0.739	< 0.001	0.542	0.023
10 s	F	0.0169	0.630	< 0.001	0.413	0.189
	CT	0.0181	0.779	< 0.001	0.326	0.204

Mean change was calculated as SD–NS. Sleep-condition comparisons used two-sided paired permutation tests in 64 participants, and associations with PVT slowing used Spearman correlations in 28 participants. Analyses covered both feature families, all three bands, and all three ROIs; only theta power variability results for the frontal and centro-temporal ROIs are shown. FDR correction was applied across the nine band-by-ROI comparisons within the power variability family for each epoch length. F, frontal; CT, centro-temporal.

More importantly, the theta power variability–PVT associations were highly consistent across the 2-, 4-, and 8-s epochs. In the frontal ROI, the correlations were ρ = 0.515, 0.544, and 0.515 for the 2-, 4-, and 8-s epochs, respectively; the corresponding correlations in the centro-temporal ROI were ρ = 0.532, 0.558, and 0.542. All six associations survived FDR correction, and the primary 4-s analysis yielded the strongest association in both ROIs. For the 10-s epochs, the associations remained positive but were weaker and did not survive FDR correction (Table 7).

Overall, both the group-level increase in theta power variability and its association with PVT slowing were preserved across multiple epoch lengths, indicating that the principal findings were not dependent on the specific use of 4-s epochs. The attenuation observed with 10-s epochs may reflect the reduced number of epoch-wise observations available for estimating variability, together with greater temporal averaging introduced by longer windows, which may reduce sensitivity to shorter-term fluctuations. These findings support the robustness of the primary results while also indicating that the strength of the variability–behavior association depends partly on the temporal scale at which variability is estimated.

### 3.4 Sex-related analyses of sleep deprivation responses

We conducted exploratory sex-related analyses of oscillatory changes, vigilance changes, and EEG–PVT associations. EEG comparisons included the full EEG sample, whereas analyses involving PVT were restricted to participants with valid behavioral data and were interpreted cautiously because of the smaller subgroup sizes.

**Figure 7:**
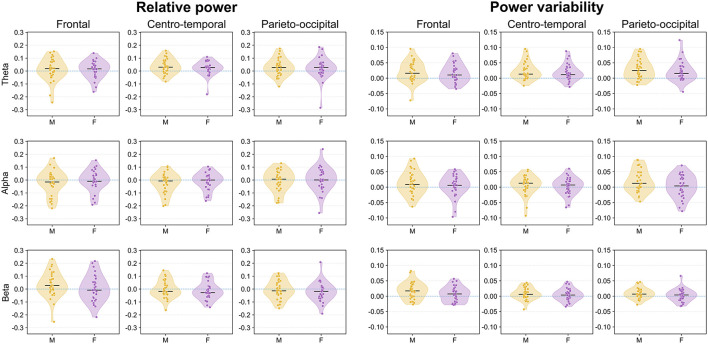
Sex-stratified distributions of sleep deprivation-related oscillatory changes. Violin plots show changes in relative power and power variability in males and females across frequency bands and ROIs. Dots represent individual participants, and dashed horizontal lines indicate zero change.

The distributions of ROI-level oscillatory changes largely overlapped across frequency bands and ROIs (Figure 7), and no sex-related differences survived within-family FDR correction in either feature family. Descriptively, males showed larger changes in parieto-occipital alpha power variability and in frontal and parieto-occipital beta power variability, although none of these differences reached uncorrected significance.

**Figure 8:**
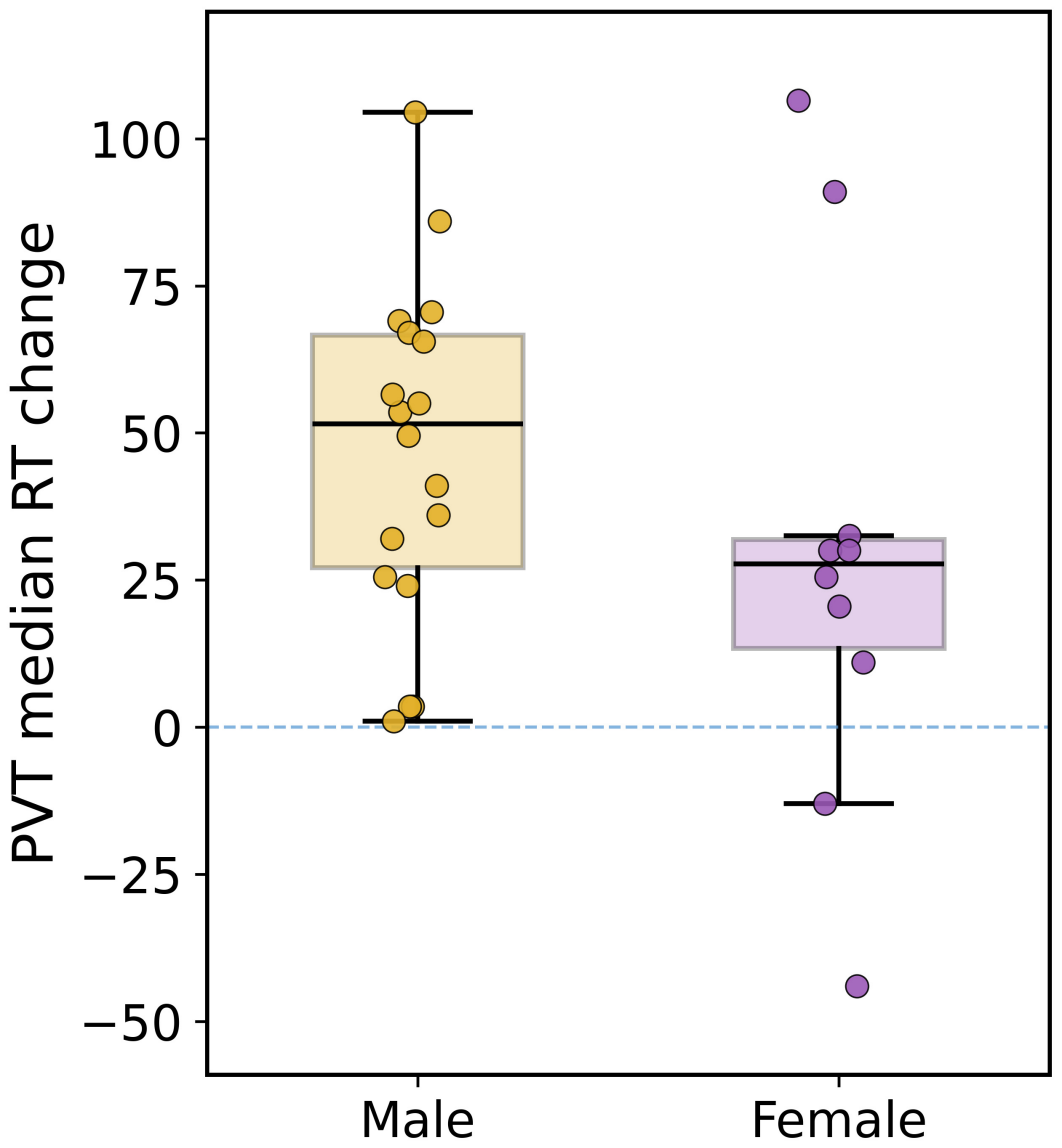
Sex-stratified changes in vigilance after sleep deprivation. Box plots show SD–NS changes in PVT median reaction time in males and females, with dots representing individual participants. The dashed horizontal line indicates zero change.

We compared SD–NS changes in PVT median reaction time between male and female participants using a median-difference permutation test and reported Cliff's delta as the effect size (Figure 8). Median increases were 51.50 ms in males and 27.75 ms in females. The between-sex median difference of 23.75 ms was not significant (*p* = 0.153), while Cliff's delta indicated a small-to-moderate effect toward greater PVT slowing in males (δ = 0.317).

**Figure 9:**
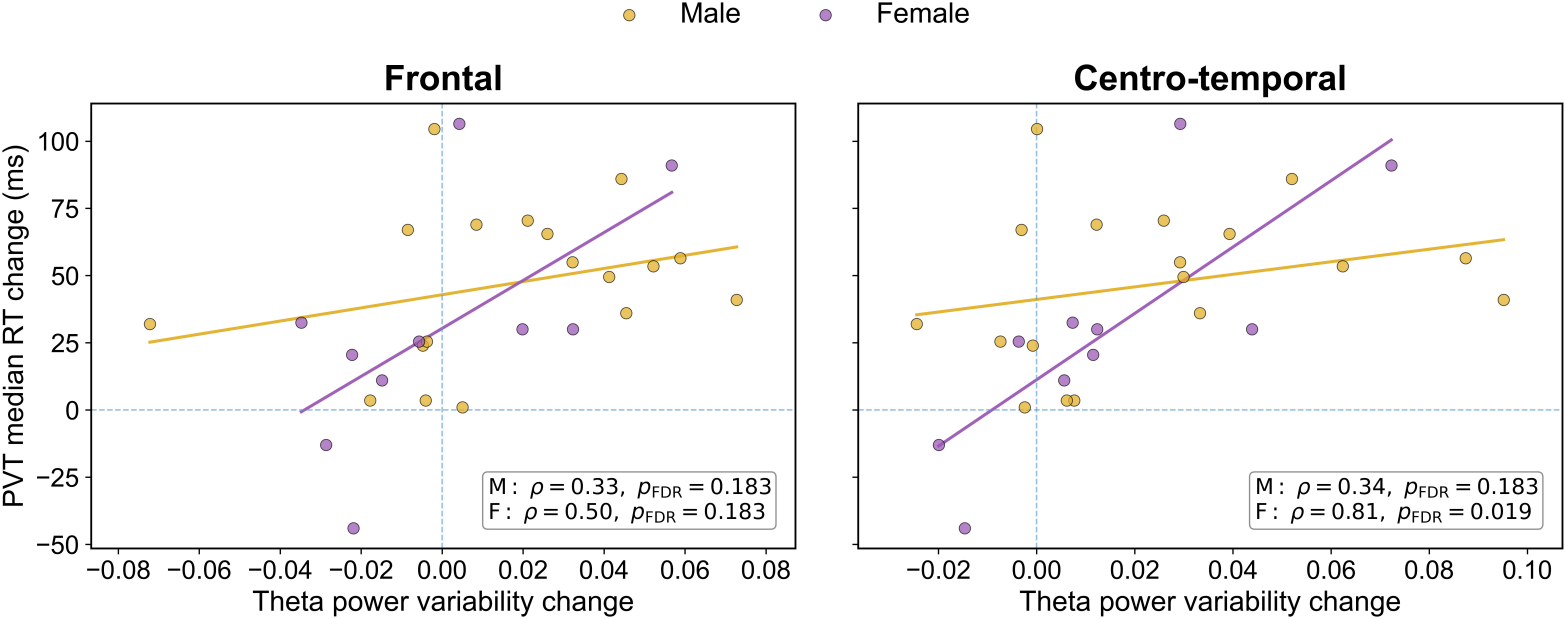
Sex-stratified associations between theta power variability changes and vigilance decline. Scatter plots show the associations between SD–NS changes in theta power variability and SD–NS changes in PVT median reaction time in the frontal and centro-temporal ROIs. Solid lines indicate sex-specific ordinary least-squares linear fits. Reported statistics are Spearman's ρ and FDR-adjusted *p* values.

Sex-stratified Spearman correlations were calculated for the two theta power variability–PVT associations that survived FDR correction, and the sex-specific patterns are shown in Figure 9. In the frontal ROI, positive correlations were observed in females (ρ = 0.50, uncorrected *p* = 0.143, FDR-adjusted *p* = 0.183) and males (ρ = 0.33, uncorrected *p* = 0.183, FDR-adjusted *p* = 0.183). In the centro-temporal ROI, the correlation was numerically stronger in females (ρ = 0.81, uncorrected *p* = 0.005, FDR-adjusted *p* = 0.019) than in males (ρ = 0.34, uncorrected *p* = 0.172, FDR-adjusted *p* = 0.183). The fitted regression line was also steeper in females, particularly in the centro-temporal ROI. We therefore formally tested sex differences in Spearman correlation strength and linear EEG–PVT slopes.

Permutation tests showed that the between-sex differences in Spearman correlations were not significant after FDR correction across the two ROIs. The difference was small in the frontal ROI (Δρ = 0.17, uncorrected *p* = 0.473, FDR-adjusted *p* = 0.473). A larger difference was observed in the centro-temporal ROI, but it did not survive FDR correction (Δρ = 0.47, uncorrected *p* = 0.089, FDR-adjusted *p* = 0.178).

The interaction between sex and the change in theta power variability was not significant in the frontal ROI (uncorrected *p* = 0.135, FDR-adjusted *p* = 0.135), but it was significant in the centro-temporal ROI after FDR correction across the two ROIs (uncorrected *p* = 0.021, FDR-adjusted *p* = 0.042). This interaction indicated different linear EEG–PVT slopes between males and females in the centro-temporal region. However, the sex-related findings remain preliminary because the corresponding difference in Spearman correlation strength was not significant and the female subgroup was small.

## 4 Discussion

This study examined sleep deprivation-related changes in EEG oscillatory features and their associations with individual differences in vigilance decline. Although sleep deprivation was accompanied by broad alterations in EEG oscillatory activity, the most informative pattern across the present analyses was observed for theta power variability. This feature showed robust sleep deprivation-related changes and, more importantly, larger increases in theta power variability were associated with greater PVT slowing, particularly in the centro-temporal region and, to a lesser extent, the frontal region. These findings suggest that the effect of sleep deprivation may not be fully captured by average changes in oscillatory power alone. Instead, increased variability of theta-band relative power may reflect reduced temporal stability of wakeful brain activity, which could be relevant to individual vulnerability to vigilance impairment. Exploratory sex-related analyses did not provide robust evidence for broad sex differences in EEG or PVT changes, but the focused analysis of theta variability-PVT associations suggested a possible sex-related pattern that requires validation in larger and more balanced samples.

### 4.1 Sleep deprivation-related changes in EEG oscillatory activity

Snipes et al. (2023) investigated changes in waking EEG oscillations during extended wakefulness and found marked alterations in theta- and alpha-band activity after 24 h of sustained wakefulness, indicating that waking EEG oscillations are sensitive to increased time awake and sleep need. Gibbings et al. (2021) further examined EEG activity, subjective sleepiness, and PVT performance after a night of mild sleep restriction and reported increased sleepiness, impaired vigilance, and changes in EEG spectral activity across frequency bands related to arousal. In keeping with these studies, the present study also observed prominent theta-related EEG changes after sleep deprivation, suggesting that increased low-frequency activity may be an important neural manifestation of elevated sleep pressure and reduced vigilance.

However, mean power only reflects the average level of activity within a given frequency band over the recording period and may not fully capture the temporal dynamics of EEG activity. Prior EEG studies have used several approaches to characterize neural variability and temporal dynamics beyond mean spectral power, including EEG microstate analysis, entropy- or complexity-based measures, and epoch-level variability metrics (Haydock et al., 2025; Lau et al., 2022; Angulo-Ruiz et al., 2021). For example, Angulo-Ruiz et al. (2021) directly examined spontaneous EEG power variability across epochs using the mean, standard deviation, and coefficient of variation of spectral power, showing that variability-based measures can complement conventional analyses of mean EEG power. Building on this perspective, the present study focused specifically on the across-epoch variability of band-limited relative power, which reflects the temporal stability of the proportional contribution of a given frequency band to the total 4–30 Hz oscillatory activity.

To operationalize this temporal stability, the present study quantified the dispersion of epoch-wise relative power using its standard deviation across epochs. This measure was selected because the study specifically aimed to characterize the magnitude of across-epoch fluctuations. Standard deviation provides a direct and readily interpretable measure of dispersion on the same scale as relative power and does not require additional parameter choices. Other temporal variability measures characterize related but distinct properties. The coefficient of variation expresses dispersion relative to the mean, whereas the variance of log-transformed power emphasizes proportional fluctuations (Angulo-Ruiz et al., 2021). Temporal autocorrelation reflects temporal persistence (Çatal et al., 2024), while entropy-based measures, including sample entropy, characterize irregularity or complexity (Lau et al., 2022). Spectral slope variability additionally captures temporal changes in the aperiodic component of the power spectrum (Wilson et al., 2022; Ameen et al., 2025). These measures are therefore complementary rather than interchangeable with the standard deviation-based measure used in the present study.

Therefore, the increase in theta power variability observed in the present study may be interpreted as greater instability of the relative contribution of theta activity across epochs after sleep deprivation, rather than merely an increase in mean theta level. Andrillon et al. (2021) found that spatially and temporally localized sleep-like slow waves during wakefulness were associated with behavioral attentional lapses and could occur while individuals were still behaviorally awake. This offers a possible explanation for the present findings: after sleep deprivation, the brain may not maintain a stable wakeful state, but may instead fluctuate between relatively stable wakefulness and transient low-alertness or sleep-like states, thereby leading to increased theta power variability.

The comparatively strong theta-band effects are unlikely to be explained solely by an overall increase in mean theta relative power. Mean theta relative power increased significantly only in the centro-temporal and parieto-occipital ROIs, whereas theta power variability increased across all three ROIs, including the frontal region. Moreover, the association between theta power variability and PVT slowing was attenuated but remained positive after controlling for mean theta relative power, particularly in the centro-temporal ROI. These findings suggest that mean theta relative power and theta power variability share variance, while variability may capture an additional temporal aspect of sleep deprivation-related theta activity. Waking theta activity is known to increase with prolonged wakefulness and has been associated with increased sleep propensity (Snipes et al., 2022, 2023). Accordingly, one possibility is that mean theta relative power reflects the overall enhancement of sleep-pressure-related low-frequency activity, whereas theta power variability reflects the uneven or intermittent expression of such activity across the recording. This interpretation remains tentative because the present variability measure does not directly identify sleep-like events or quantify sleep pressure.

### 4.2 Theta power variability and vigilance decline

Sleep loss can impair vigilant attention through a general slowing of typical responses, in addition to episodic attentional failures. DiFrancesco et al. (2019) found that restricted sleep increased PVT median reaction time. Raison et al. (2025) further distinguished the general slowing of typical responses from periods of unresponsiveness during prolonged wakefulness. Temporal instability in neural oscillatory activity may be relevant to this general response slowing. Yu et al. (2024) found that EEG spectral-power volatility predicted problem-solving success and solution time, suggesting that the temporal stability of oscillatory activity is related to cognitive performance. At the neurophysiological level, Akella et al. (2025) identified distinct oscillatory states in the mouse visual cortex that differed in neuronal variability and sensory encoding, providing evidence that cortical information processing changes dynamically across internal oscillatory states. In light of these findings, greater theta power variability after sleep deprivation may reflect a reduced capacity to maintain a stable and efficient wakeful processing state, potentially contributing to the overall slowing of typical responses reflected by increased PVT median reaction time.

More broadly, theta power variability can be situated within the literature on time-varying neural activity. EEG microstate analysis characterizes rapid transitions between quasi-stable whole-scalp topographic configurations (Haydock et al., 2025), whereas dynamic functional connectivity and metastability-based approaches examine time-varying interregional coordination and transitions among large-scale network states (Cho et al., 2024; Hancock et al., 2025). Temporal analyses of oscillatory networks similarly emphasize that neural rhythms fluctuate across time rather than remaining stationary throughout a recording (Akella et al., 2025). In comparison, the measure used here provides a simpler description of oscillatory dynamics by quantifying the dispersion of theta relative power across epochs. Because it summarizes fluctuation magnitude using the across-epoch standard deviation, it does not explicitly retain the temporal ordering of epochs, identify recurring spatial states, or characterize changes in interregional connectivity. Thus, the observed increase in theta power variability may reflect reduced temporal stability of theta-band activity, but it should not be considered a direct measure of neural metastability or dynamic network reconfiguration. Future studies combining these approaches may clarify whether they capture shared or complementary aspects of sleep deprivation-related neural dynamics.

Evidence from individual-vulnerability studies further suggests that theta-related responses to sleep loss vary across individuals. Chua et al. (2014) classified participants as vulnerable or resilient to sleep deprivation and found that vulnerable individuals showed slower and more variable PVT responses, together with higher and more variable waking theta-band EEG power. More recently, Zhang et al. (2025) reported larger changes in frontal theta power among individuals vulnerable to alertness decline during overnight wakefulness, whereas resilient individuals maintained relatively stable resting-state EEG activity. Although these studies examined theta dynamics over different temporal scales, both indicate systematic inter-individual differences in theta-related responses to sleep loss. The present study extends this evidence by demonstrating a continuous association between sleep deprivation-related increases in within-recording theta power variability and increases in PVT median reaction time, thereby extending categorical vulnerability comparisons to a continuous individual-differences framework. Theta power variability may therefore provide a continuous indicator of individual vulnerability to vigilance decline after sleep deprivation.

Consistent with this interpretation, the follow-up partial correlation analysis suggested that theta power variability was related to, but not identical with, mean theta relative power. The association between variability and behavior was attenuated after controlling for mean theta relative power, indicating that the two measures share variance. However, the remaining positive association, particularly in the centro-temporal ROI, suggests that variability may capture partially non-redundant information about vigilance decline. Therefore, theta power variability should be interpreted as a complementary feature of theta activity rather than as a fully independent marker.

The aperiodic-adjusted analysis provided further evidence that the observed theta-band effects were not solely driven by changes in the broadband spectral background. After removal of the fitted aperiodic component, theta-band residual power and residual variability remained elevated after sleep deprivation across the three ROIs. However, the association between theta-band residual variability and PVT slowing remained significant only in the centro-temporal region, whereas the corresponding frontal association was attenuated. This pattern suggests that the centro-temporal theta variability–vigilance relationship was particularly robust to aperiodic adjustment, while the frontal association may have partly reflected shared variance with broader spectral changes. Nevertheless, because spectral parameterization represents a model-based decomposition of the power spectrum, the residual features should not be interpreted as completely isolated or physiologically pure oscillatory activity (Donoghue et al., 2020; Wilson et al., 2022).

### 4.3 Spatial distribution and neural implications

Given that the PVT requires sustained vigilance and rapid responses, the regional distribution of the EEG-PVT associations may be functionally meaningful. Frontal regions are primarily involved in sustained attention, cognitive control, and response selection. Dong et al. (2024) found that total sleep deprivation altered EEG effective connectivity from the left cuneus to the right middle frontal gyrus, with greater connectivity changes associated with larger increases in PVT reaction time. Yao et al. (2023) also reported that sleep deprivation-related resting-state functional alterations in the bilateral middle frontal gyri were associated with greater slowing in PVT mean reaction time. Although these studies examined neural measures different from theta power variability, their findings collectively suggest that frontal functional alterations after sleep deprivation may be related to impaired sustained vigilance and rapid responding.

The centro-temporal scalp region is broadly involved in sensorimotor integration, sensory processing, and response-related activity. Gorgoni et al. (2014) found that sleep deprivation-related increases in theta activity, mainly over centro-posterior scalp areas, were associated with slowing of the fastest PVT responses and increased subjective sleepiness, and this spatial distribution partially overlaps with the present centro-temporal pattern. Ma et al. (2023) further suggested that total sleep deprivation may impair vigilance and sensory information processing within the sensorimotor network. Therefore, greater theta power variability over centro-temporal scalp sites may reflect reduced stability of neural processes supporting vigilant responding and sensorimotor information processing after sleep deprivation.

Notably, theta power variability remained significantly associated with PVT slowing at the channel level. Among the seven electrodes that survived FDR correction, the adjacent F5 and FC5 sites showed the strongest associations. Previous studies have linked frontal and frontocentral theta activity to cognitive control, response selection, and the adjustment of action plans during motor preparation and execution (Pellegrino et al., 2018; Eisma et al., 2021). In the PVT used in the present study, participants were required to detect the appearance of a visual target and press a response button as quickly as possible. Therefore, this spatial pattern suggests that reduced stability of frontal–frontocentral theta activity after sleep deprivation may reflect instability in the neural state of response and execution.

Overall, the ROI-level findings were consistent with the finer spatial localization provided by the channel-level analysis, with both pointing to the frontal and centro-temporal regions. This consistency suggests that increased theta power variability in these regions after sleep deprivation may reflect reduced stability of the overall neural functional state supporting vigilant responding, motor preparation and execution, which may in turn be expressed behaviorally as a general prolongation of PVT reaction time.

### 4.4 Exploratory sex-related patterns

The sex-related analyses provided additional exploratory context for the inter-individual differences observed in this study. Previous studies have suggested that the effects of sleep deprivation on behavioral performance may show sex-related differences, although the findings have not been fully consistent (Hajali et al., 2019; Spitschan et al., 2022). For example, Ołpińska-Lischka et al. (2020) noted that studies on sex differences in psychomotor performance after sleep deprivation remain limited and that existing findings are somewhat inconsistent. Against this mixed background, the present study did not find robust evidence for sex differences in sleep deprivation-related EEG changes or PVT slowing, suggesting that the main EEG alterations and behavioral impairment were unlikely to be driven solely by male–female differences in the average magnitude of responses.

Further focusing on the main EEG-PVT findings, the sex-stratified correlation analyses showed numerically stronger associations in females, particularly in the centro-temporal region. However, the between-sex difference in Spearman correlation strength was not significant after FDR correction and should not be interpreted as definitive evidence of a sex difference in correlation strength. Separately, the sex theta power variability interaction was significant in the centro-temporal region, indicating a sex-related difference in the linear association between theta power variability changes and PVT slowing. Specifically, females showed a steeper fitted slope, suggesting that a given increase in theta power variability may correspond to greater behavioral slowing in females than in males. This pattern is compatible with broader evidence that cognitive responses to sleep loss may differ by sex (Hajali et al., 2019). However, given the small and unbalanced EEG-PVT subsample, this finding should be regarded as exploratory. Future studies with larger and more balanced samples are needed to determine whether the association between theta power variability and PVT slowing differs consistently between males and females.

### 4.5 Limitations and future directions

Several limitations should be considered. First, the effective behavioral sample size was relatively small, as only 28 participants had valid PVT data under both normal sleep and sleep deprivation conditions. This limited the statistical power of the EEG–PVT association analyses and was particularly relevant to the exploratory sex-related analyses, which were based on small and unbalanced subgroups. Therefore, the sex-related findings require validation in larger and more balanced samples. Second, the behavioral analysis focused on PVT median reaction time, which reflects overall response slowing but does not capture attentional lapses or reaction time variability. Therefore, it remains unclear whether theta power variability is specifically related to response slowing or more broadly to vigilance impairment. Third, the ROI scheme was based on broad scalp regions, which may have obscured finer spatial patterns despite the complementary channel-level analyses. Fourth, because the EEG–PVT associations were correlational and based on SD–NS change scores, they cannot establish a causal relationship between theta power variability and vigilance decline. Fifth, because EEG was recorded during resting state rather than during PVT performance, the generalizability of the present findings to task-related EEG remains uncertain. Resting-state EEG may reflect background neural alterations associated with vigilance decline after sleep deprivation, but it does not directly capture neural dynamics elicited during active task performance (Çatal et al., 2024). Therefore, it remains to be determined whether similar theta power variability patterns and EEG–PVT associations are observed in task-related EEG. Finally, the use of an existing public dataset constrained the available behavioral measures, sample composition, and experimental details.

Future studies should validate the present theta variability–PVT findings in larger independent samples with more complete behavioral data and more balanced sex distributions. Future work should also clarify how theta power variability relates to conventional spectral power measures, including mean relative power and absolute power-based features, as these measures may capture different aspects of sleep deprivation-related oscillatory changes. Broader behavioral outcomes, such as PVT lapses, response speed, and reaction time variability, should be examined to determine whether theta power variability is specifically associated with vigilance slowing or more broadly related to sleep deprivation-related impairments in sustained attention. Future studies combining resting-state and task-related EEG within the same participants are needed to determine whether theta power variability reflects a general neural marker of vulnerability to sleep deprivation or is specific to the resting-state condition.

## 5 Conclusions

In this study, we examined sleep deprivation-related changes in EEG oscillatory features and their associations with vigilance decline. Sleep deprivation was accompanied by changes in relative power and power variability, with theta power variability showing the most consistent pattern across the present analyses. Participants with larger increases in theta power variability tended to exhibit greater slowing in PVT median reaction time, particularly in the frontal and centro-temporal regions. These findings provide insight into the relationship between the temporal variability of EEG oscillatory activity and behavioral impairment after sleep deprivation and may help characterize individual differences in vigilance decline. Future studies should examine these associations in larger and more balanced samples and determine whether variability-based EEG measures can reliably characterize sleep deprivation-related vigilance impairment.

## Data Availability

The original contributions presented in the study are included in the article/supplementary material, further inquiries can be directed to the corresponding author.
